# Movement as therapy an analytical study of the role of physical activity in building strong mental health for older adults

**DOI:** 10.3389/fpubh.2026.1797406

**Published:** 2026-04-10

**Authors:** Anwar Al-Nuaim, Ahmed K. Hassan

**Affiliations:** Department of Physical Education, College of Education, King Faisal University, Al-Ahsa, Saudi Arabia

**Keywords:** anxiety, depression, healthy aging, mental health, older adults, physical activity, quality of life, stress

## Abstract

**Background:**

Healthy aging and community well-being are the priorities of Egypt Vision 2030; hence, the need to promote non-pharmacological approaches to mental health among the older adults. This paper examines how physical activity can be used as a form of therapy in improving the mental health and quality of life of older adults who attend sports clubs in EL Minya, Arab Republic of Egypt, using the notion of movement as therapy.

**Methods:**

A cross-sectional, quantitative research design was used, and a sample of 422 older adults (aged ≥60 years) was used. The participants took a questionnaire in a structured form (Arabic version of DASS-21 to evaluate depression, anxiety, and stress) and the Rapid Assessment of Physical Activity (RAPA). The data were reviewed on descriptive statistics, chi-square tests, and multiple linear regression to determine the relation between the level of physical activity and mental health outcomes, and the results were adjusted by age.

**Results:**

It was found that almost one-third (31) of the respondents did not have any physical activity, and the remaining 69.2% participated in different levels of physical activity weekly. The most common was walking (92.4%). The level of mental health symptoms was also much lower in the highly active group; that is, individuals who exercised 10 h/week or more had a mean DASS-21 of 12.3 (normal) versus 34.2 among inactive ones (clinically significant distress). The relationship between dose and response was evident (all *p* < 0.001).

**Conclusion:**

The level of exercise was the strongest negative predictor of psychological distress (*β* = 0.41, *p* < 0.001) and could predict 23 percent of the variance of the DASS-21 scores (*F* = 31.28, *p* < 0.001). The results justify the inclusion of customized and socially entrenched movement initiatives, especially walking and light aerobic activities, in national aging and mental health policies. This initiative can make physical activity not a personal habit but a civic intervention, both dignified, resilient, and mentally healthy aging, in accordance with Egypt Vision 2030.

## 1 Introduction

There is an immense demographic shift taking place in the world, and by 2050, the number of people over 60 years old will pass 2 billion, which is almost twice the number of 2015 (1, 2). The phenomenon of this severe aging is a multifaceted problem that touches upon health, social unity, and the state of mind. The older adults are specifically susceptible to psychological illnesses, such as depression, anxiety, and dementia, which are usually complicated by the aging process and social segregation (3). This forces the dire need to find efficient, non-pharmacological interventions that improve quality of life and active non-pharmacological aging. In addition, novel care designs and technologies, such as telehealth and wearable devices, can support the management of health in a better way and encourage independence in the older adults (1, 4). It is important to tackle these issues by implementing policies that are wide-ranging and interdisciplinary to have an active and fulfilling life among older adults (4).

Physical exercise has been identified as one of these interventions that have become the foundations of prevention and treatment measures. The existing body of evidence of the world testifies to the fact that active sports can greatly lower depression and anxiety symptoms, improve mood and life satisfaction, and even help to maintain cognitive abilities in old age (5, 6). Systematic reviews also attest to the fact that physical activity may be as useful as psychotherapy or medication in the treatment of some mental disorders (7, 8). In addition to the psychological advantages, physical exercise reduces physiological age-associated aging, which makes the biological age younger and strengthens the psychological resilience (5, 9). More importantly, the positive effects of exercise do not pertain to an individual level but to social. Group-based activities (walking, tai chi, dance) not only enhance physical capacity but also counter loneliness and sense of belonging to the community, which are critical in psychosocial well-being (10–12). Meta-analyses are in favor of these multifaceted gains and significant positive alterations in physical health and social interaction are noted (13). Although such convincing, older adults still do not participate in exercise because of such barriers as chronic illness, lifelong sedentary behavior, lack of motivation, fear of falling, and negative aging stereotypes (14–16). The COM-B model also offers capability, opportunity, and motivation as the key determinants of engagement that are hindered by environmental, cultural, and perceptual confines (17).

Nevertheless, there is still a gap in critical research. Although the health advantages of physical activity on mental health among older adults populations are widely reported in the world literature, much is unknown regarding the therapeutic advantages of physical activity among older adults in Egypt and the Arab world at large. Available literature on the region has mostly involved youth or overall physical health data (18), and little has been considered on the dose–response association of structured movement and psychological distress in the aging population. This is especially alarming considering the fast aging of the Egyptian population and the increasing levels of depression, anxiety, and social isolation among the older citizens of the country. Therefore, the proposed study will cover a pressing national concern in accordance with Egypt Vision 2030: to find available, culturally appropriate, non-pharmacological methods of healthy aging. We also examine the hypothesis that increased regular physical activity is also strongly related to reduced levels of depression and anxiety and an increase in self-reported quality of life in older adults in Egypt. This study will provide policy-makers, healthcare professionals, and community organizations with localized empirical information to shape their own wellness programs that include a component of movement as therapy into national geriatric care systems—ultimately leading to dignity, autonomy, and mental health within the aging Egyptian population (19).

### 1.1 Research questions

Q1: How common is self-reported physical activity in older adults?

Q2: In which condition is the mental health of such a population (depression, anxiety, perceived stress, etc.)?

Q3: Are there any statistically significant differences between older adults concerning the levels of physical activity and the results of mental health (depression, anxiety, and stress)?

### 1.2 Literature review

The role of physical activity in increasing mental well-being and quality of life among older adults is supported by the multifaceted nature of this phenomenon, which is reflected in its literature. Physical exercise is very important to the older adults, and very few of them adhere to the prescribed standards, which indicates the necessity of effective programs aimed at enhancing the number of participants (20). Physical exercise and physiotherapy have been revealed to decrease depressive and anxiety symptoms, enhance cognition, and increase functional autonomy, memory and socialization in the older adults (21). It is proven that different types of physical activities like functional training, water exercises, dance, and yoga are helpful in ways of preserving mobility and mental health and need to be provided with access to such activities (22). Physical activity significantly impacts the quality of life by promoting physical, mental, spiritual, and social health, which are essential for a fulfilling old age (23). Despite these benefits, there is a call for more standardized protocols and long-term studies to better understand the impacts of these interventions and to address barriers to physical activity among older adults (21).

The interaction of physical activity with older adult health has become an issue of growing attention, as its impacts on physical and mental health and quality of life have been proved. One of the world’s pieces of evidence indicates that daily exercising even in their simplest forms is linked to a reduced incidence of depression and anxiety, better cognitive abilities, and a higher rate of overall life satisfaction (5). According to a recent systematic review, group physical activities with a combination of physical and social aspects have a multiplier effect on the improvement of the quality of life in older adults (11). Mental health disorders also have exercise as an effective preventive and therapeutic intervention; therefore, it is an important part of non-pharmacological mental health interventions (9). The physical aspect of Egypt needs to encourage the older adults to exercise. Research findings suggest that about 60 percent of middle-aged and older people are not physically active. Although this group mostly engages in physical exercise, it is not always performed at a level and frequency to bring about optimal psychological and physical advantages (24). The other literature indicates that overweight sedentary lifestyles are an emerging national health issue that affects all age groups, with older people being the most affected, with a higher risk of experiencing physical impairments and social alienation (25). Although much of the local research in the Arab world has involved youth and students in terms of the relationship between physical activity and mental health (17), there is some growing evidence that argues that the relationship may be related to the older population as well. The investigation of older Egyptian revealed that there is a correlation between the level of physical activity and cardiovascular health, which is commonly associated with mental health (18). The recent qualitative research is investigating the issues impacting the engagement of older Saudis in physical activities, including fear of falls, inappropriate facilities, and cultural or family constraints (14, 17), which may be aggravated without specific interventions in the Egyptian context.

Moreover, researchers in the Arab world and the Gulf region, including Malaysia, have found that the correlation between physical activity and quality of life does not necessarily maintain statistical significance in all dimensions, especially in the dimensions concerning independence and financial status; it has a stronger relationship in the social and emotional dimensions (10). This indicates that the therapeutic effect of movement can be better reflected in enhancing social relationships and sense of belonging, which are important aspects in Arab culture, than its primary impact on economic or physical indicators. So, it is possible to say that another applied study in the context of Egypt needed to comprehend the character of the connection between physical activity, mental health, and quality of life in older adults with references to cultural and social peculiarities. The development of efficient programs to advocate the concept of movement as therapy involves a comprehensive comprehension of the demands, obstacles, and incentives of this population within Egyptian society, and that is what this research intends to add to.

## 2 Materials and methods

Data were gathered on older adults using a cross-sectional study design through self-administered questionnaires with the help of trained staff during a period of 3 months in older adults in public and private sports clubs in Elminya, Arab Republic of Egypt. The age of all the persons who attended these clubs on a regular basis and were above 60 years of age was eligible to participate. Participants who had severe cognitive impairment (according to club staff or caregivers) or had acute medical conditions that disallowed physical activity were not included in the study. The target population was completely reachable and recognizable, leading to the final sample of 422 older adults and the total cohort of people eligible in the selected clubs during the data collection time. Data collection was done using a structured, self-administered questionnaire in Arabic (primary) and English (supplementary) languages. The questionnaire was self-administered, but trained enumerators were available to support participants who had literacy problems or visual challenges. Items are read aloud by enumerators and not interpreted with the aim of ensuring that they are understood and the rate of response bias is reduced. The questionnaire has been divided into three major areas: In section 1, there were socio-demographic and lifestyle variables such as age, gender, marital status, level of education, the presence of chronic diseases (e.g., diabetes, hypertension, arthritis), daily internet use (none, less than 1 h, 1–3 h, more than 3 h), and self-reported general physical health status (excellent, very good, good, fair, poor) (see Table 1).

**Table 1:** Demographic characteristics of the study sample (*N* = 422).

Characteristic	Profile	*n*	%
Gender	Male	256	60.7%
	Female	166	39.3%
Age	60–65	198	46.9%
	66–70	176	41.7%
	71–79	48	11.4%
Marital status	Married	332	78.7%
	Widowed	68	16.1%
	Divorced/separated	14	3.3%
	Single	8	1.9%
Education level	No formal education	94	22.3%
	Primary (1–6 years)	112	26.5%
	Intermediate (7–9 years)	88	20.9%
	Secondary (10–12 years)	76	18.0%
	University or higher	52	12.3%
Chronic health conditions	None	62	14.7%
	One condition	146	34.6%
	Two or more conditions	214	50.7%
Daily internet use	None	102	24.2%
	<1 h	138	32.7%
	1–3 h	124	29.4%
	>3 h	58	13.7%
Self-reported general health	Excellent/very good	96	22.7%
	Good	184	43.6%
	Fair	112	26.5%
	Poor/very poor	30	7.1%

In Section 2, The measure of physical activity was done by the Rapid Assessment of Physical Activity (RAPA), a reported 7-item scale that had been modified to suit older adults. It determines frequency, intensity, and nature of aerobic and strength activities to classify the participants into sedentary, underactive or active. In order to determine the level of physical activities among older adults, the version of the Rapid Assessment of Physical Activity (RAPA) was used, which was adapted, a self-report scale of 7 items, briefly, originally created by Almaged et al. (26), Topolski et al. (27) to evaluate physical activities among adults aged 60 years and above. To ensure the study objectives and the Egyptian cultural context, the RAPA was adapted to measure not only the categorical activity status (sedentary, underactive, active) but also assesses the estimated amount of time spent in the physical activity, in hours, which allows the doseresponse analysis of the relationships with mental health outcomes. The modified instrument had three items addressing aerobic activities (light, moderate, and vigorous), two items in strength and flexibility exercise, and a checklist of culturally specific activities (e.g., walking, light aerobics and movement) (see Appendix A). The questionnaire was translated into Arabic following the WHO direction and the quality of the questionnaire was checked by a group of three gerontology, public health, and Arabic linguistics experts to verify the content validity, understanding, and cultural fit. It was then pilot-tested on 20 older adults following minor refinements and found to be face valid and feasible. The reliability analysis generated Cronbach alpha of 0.84, which depicts a good internal consistency and the expert review and pilot feedback confirmed its construct and content validity in use by older Egyptian adults.

Section 3 evaluated the mental health outcomes with the use of Arabic version of the Depression, Anxiety and Stress Scale (DASS-21). The 5-point Likert scale (0, 1, 2, 3, and 4) is used to rate the statements provided in this scale with the scoring of 0 (did not apply at all) and 4 (applied to me very much or most of the time) being applicable on the extremes. The products are further categorized in three sub scales that are Depression (D), Anxiety (A), and Stress (S), and subtotal scores in each of the subscales are detailed in the framework of the levels of severity, such as normal, mild, moderate, severe, and the ones that are extremely severe (see Appendix B) (26). To establish the content validity of the questionnaire, the questionnaire was subject to the review of three gerontology, public health, and psychological assessment specialists. Their statements were directed at being clear, culturally and rather relevant to the scenario of Egyptian elders. Minor changes were made and reintroduced to be adopted. The pilot study had been carried out on a sample of 20 older adults of a sports club that did not constitute the main sample. The questionnaire was released to the respondents and the respondents were reinterviewed after 2 weeks in order to determine the test–retest reliability. Internal consistency and stability were estimated using Cronbachs alpha (*α*) and Cohens kappa, respectively. The correlation coefficients were above 0.75 and it demonstrated that there was a satisfactory consistency and reproducibility. The IRB of Minia University accepted the research protocol. All the participants provided informed consent in data collection. The research was complying with the ethical principles of declaration of Helsinki. To maintain confidentiality and privacy, it was a voluntary and anonymous process and the information was saved somewhere safe and anonymously analyzed.

### 2.1 Time line and statistical analysis of data

This cross-sectional research was being carried out within 3 months, i.e., June to August of 2025, in the summer season, when sport clubs in El Minya, Egypt, usually show regularity in attendance and regular operations among the older adults. It was an opportune time; the time that would enable the project to attain a stable participation and eliminate seasonal interference in physical activities. IBM SPSS Statistics version 26 (IBM Corp., Armonk, NY, USA) was utilized to analyze quantitative data, and the study included two stages: first, descriptive statistics, such as frequencies, percentages, means, and standard deviations, were used to summarize the demographic factors, physical activity levels, and mental health of the participants (measured by means of the DASS-21); second, inferential analysis was performed to assess the associations and predictive relationships, including internal consistency assessment subscales through Cronbach’s alpha.

## 3 Results

### 3.1 Physical activity

**Figure 1:**
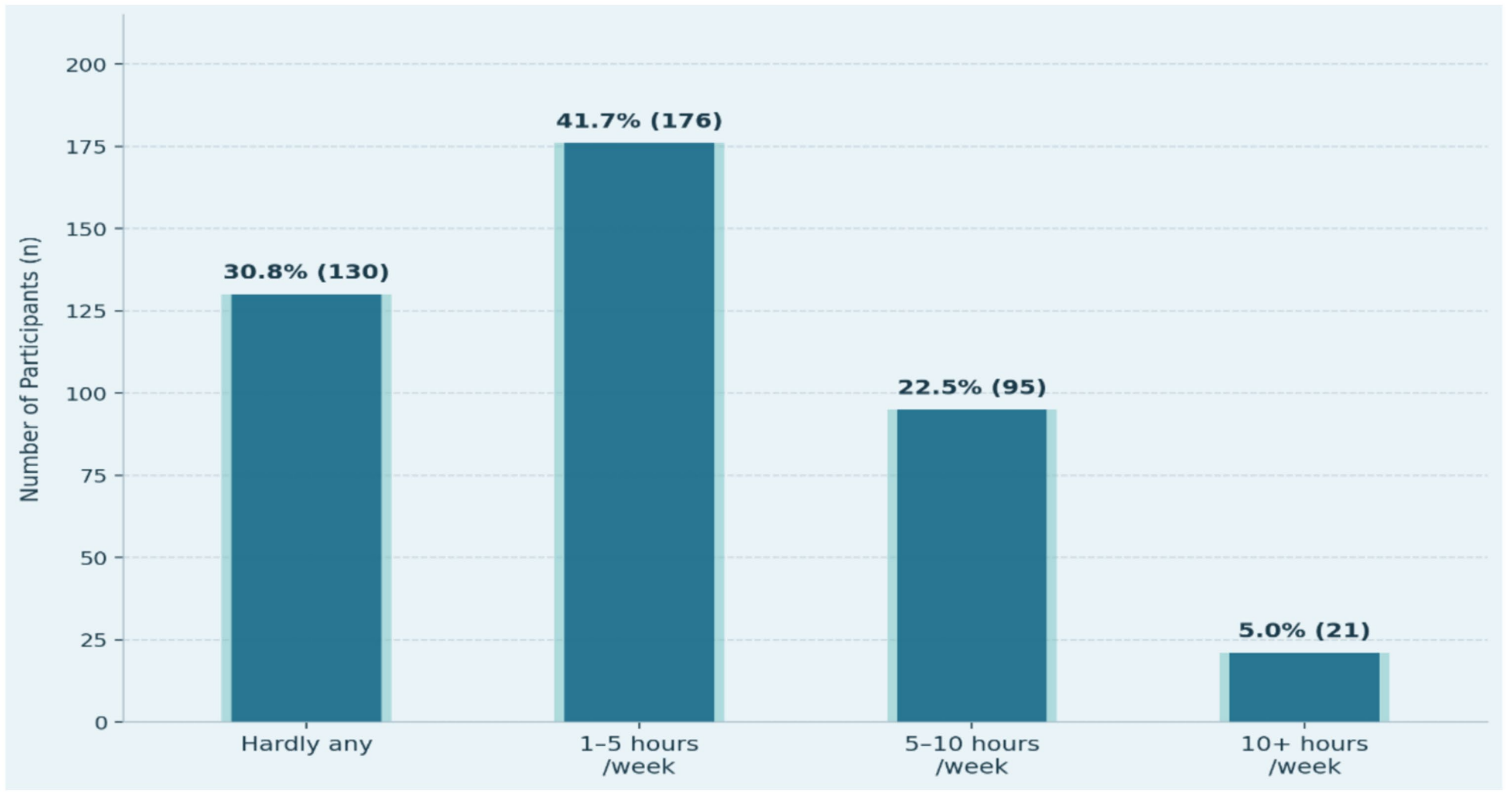
Weekly physical activity duration among older adults attending sports clubs.

Table 2 shows the physical activity levels among the older adults indicate a moderate but mixed engagement problem, as indicated in Table 2 below. Whereas 30.8% (*n* = 130) of the respondents confirmed that they were involved in hardly any physical activity, the greater number of respondents (69.2%, *n* = 292) were involved in some kind of regular activity. Particularly, 41.7% (*n* = 176) of them were asked to spend between 1 and 5 h per week, which is the biggest subgroup; 22.5% (*n* = 95) were asked to spend between 5 and 10 h per week; and 5.0% (*n* = 21) of all were asked to spend more than 10 h per week (see Figure 1). Such a distribution indicates that, although immobility is still observed in a significant proportion of the older adults population, most of those who visit sports clubs in Al-Ahsa are at least moderately physically active, which is probably due to their personal motivation as well as the surrounding conditions they have at these facilities (see Figure 1).

**Table 2:** Frequency distribution of physical activity levels among older adults (*N* = 422).

Weekly physical activity duration	*n*	%
Hardly any	130	30.8%
1–5 h	176	41.7%
5–10 h	95	22.5%
10+ h	21	5.0%

**Figure 2:**
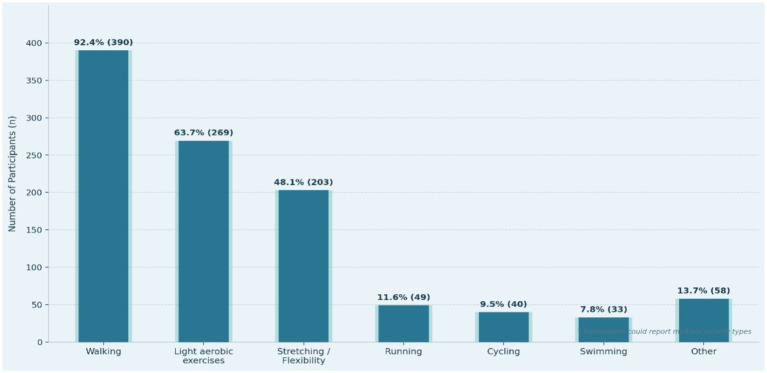
Types of physical activities practiced by older adults.

Table 3 data show that the most popular types of physical activities among older adults are those of low impact and accessible and socially integrated activities, with walking being the most common (92.4%), followed by light aerobic exercises (63.7%) and stretching/flexibility activities (48.1%), depending on safety, cultural behavior, and the presence of organized activities in sports clubs in the area. Conversely, activities that were more intense or required facilities, including running (11.6%), cycling (9.5%), and swimming (7.8%), were much less prevalent, presumably because of physical constraints, perceived risk, and unavailability of suitably designed facilities. Moreover, 13.7% said that they engaged in culturally based practices such as light farming or movement as part of everyday practice, which highlighted the importance of local circumstances in influencing exercise behavior see Figure 2. Together, these trends indicate a powerful tendency towards safe, sustainable, and socially meaningful types of movement, which contributes to the possibility that movement as therapy is successfully incorporated into the health of the population and community-based aging.

**Table 3:** Types of physical activities reported by older adults (*N* = 422).

Activity type	(*n*)	(%)
Walking	390	92.4%
Light aerobic exercises	269	63.7%
Stretching/flexibility	203	48.1%
Running	49	11.6%
Cycling	40	9.5%
Swimming	33	7.8%
Other	58	13.7%

### 3.2 Mental health (DASS)

**Figure 3:**
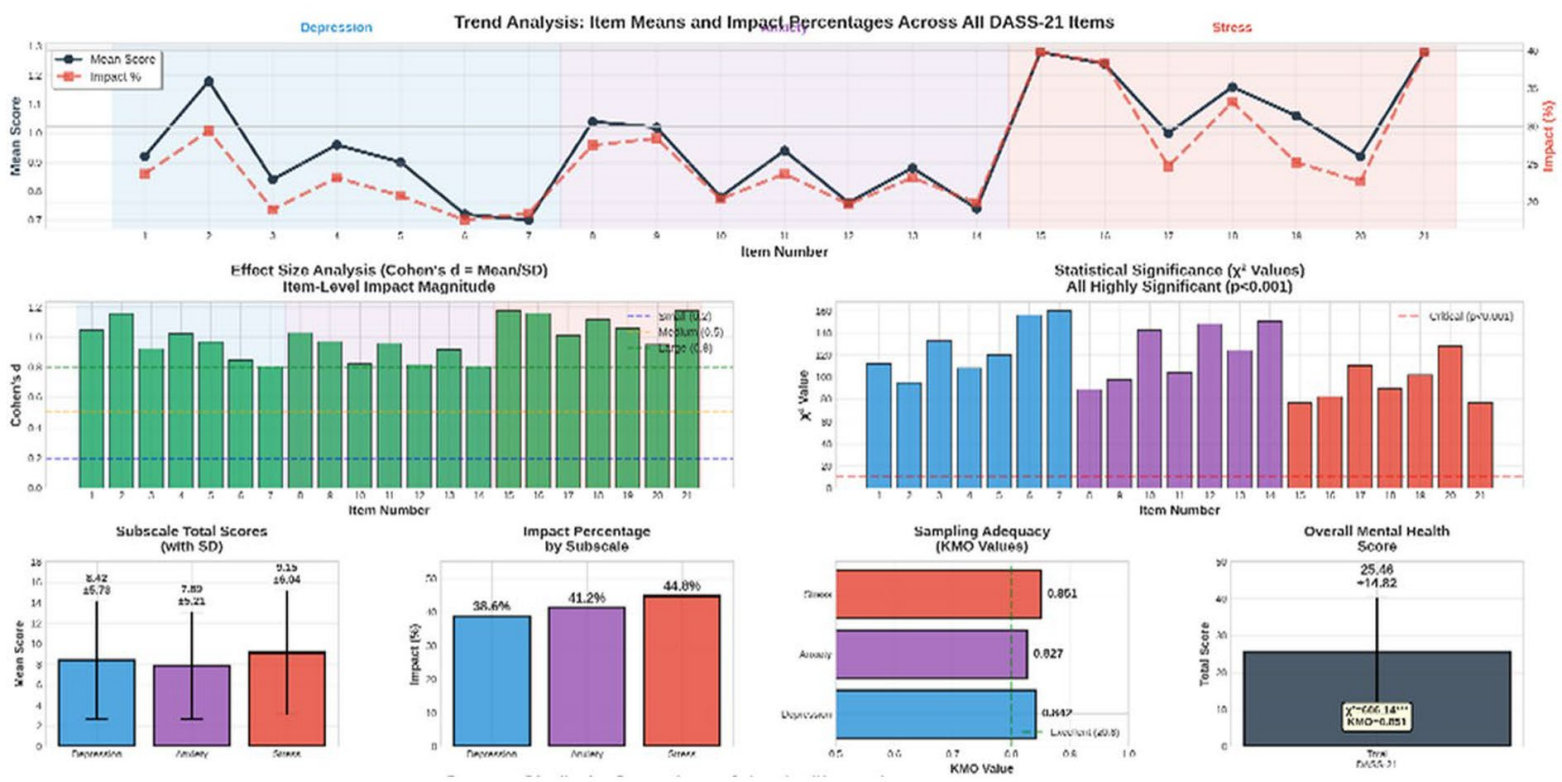
Comprehensive analysis: response distribution, effect sizes, and trends, with emphasis on the three dimensions (depression, anxiety, and stress).

Figure 3 shows a frequency distribution and psychometric properties of the responses on the DASS-21 subscales in 422 older adults. In the outcomes, although most participants mentioned a low level of endorsement of severe symptoms, a significant percentage of them reported a high level of moderate to severe psychological distress. 38.6% of respondents had moderate–severe depressive symptoms, with feelings of meaninglessness and low self-worth among the least common (impact percent = 18.5–19.0%), and difficulty in initiating activities was the most supported symptom of depression (impact percent = 29.4%). The use of anxiety symptoms was reported by 41.2% of the sample, with dry mouth and breathing difficulties being the most affected (27.528.4%), and fear-related items (e.g., panic, unexplained fear) were less supported. The most common area was stress, with the largest proportion of the respondents (44.8) having moderate–severe stress levels; the highest level of the burden of the symptoms was noted in items connected to overreacting to minor stressors and high nervous energy (impact % = 39.8). The sampling adequacy of all the subscales (KMO > 0.82) and the statistically significant chi-square values (*p* < 0.001) demonstrated that the responses were not random and that the DASS-21 had a strong internal structure in this population. The overall DASS-21 (M = 25.46, SD = 14.82) is also a strong indicator of the presence of clinically significant psychological symptoms in this group of community-residing older adults individuals (see Appendix B), and data were shown in Supplementary Table 1.

**Figure 4:**
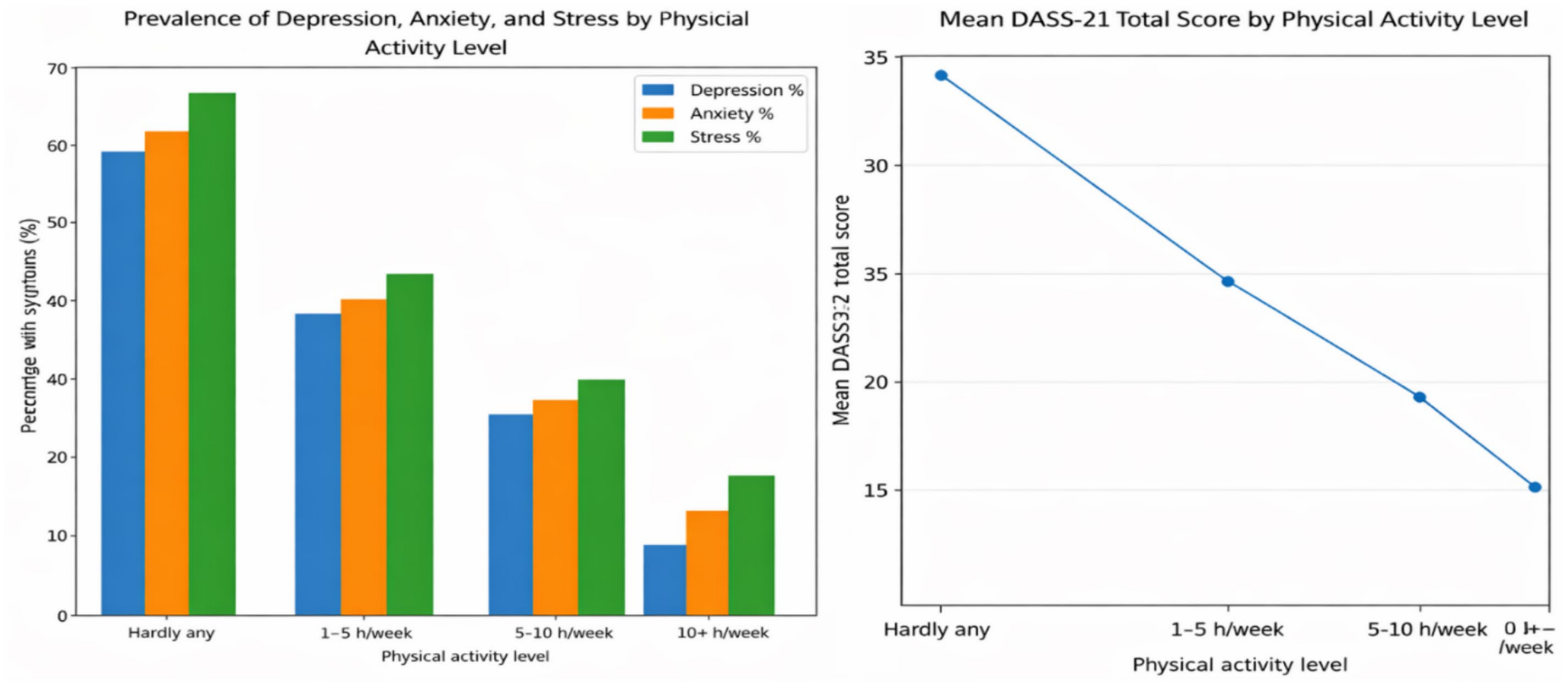
The percentage of participants experiencing depression, anxiety, and stress, as well as their overall DASS-21 score, in each physical activity category.

Table 4, Figure 4 show that there is a strong negative correlation between the levels of physical activity and the levels of mental health symptoms. The prevalence of moderate-to-severe depression (58.5%), anxiety (62.3%), and stress (67.7%) was the highest in older adults who reported hardly any physical activity, with a mean DASS-21 total score of 34.2, which is a clinically significant distress. Conversely, individuals who participated in 10 or more hours of exercise per week had significantly fewer symptoms (depression: 9.5; anxiety: 14.3; stress: 19.0) and the least overall DASS score (12.3). An evident dose–response relationship is also observed: the higher the physical activity per week, the lower the prevalence rates of psychological distress. All the correlations were significant (*p* < 0.001).

**Table 4:** Association between physical activity levels and mental health status (DASS-21) among older adults (*N* = 422).

Physical activity level	Depression % (*n*)	Anxiety % (*n*)	Stress % (*n*)	Mean DASS total
Hardly any (*n* = 130)	58.5% (76)	62.3% (81)	67.7% (88)	34.2 ± 12.1
1–5 h/week (*n* = 176)	36.4% (64)	38.6% (68)	42.0% (74)	24.8 ± 11.3
5–10 h/week (*n* = 95)	22.1% (21)	24.2% (23)	27.4% (26)	18.5 ± 9.7
10+ h/week (*n* = 21)	9.5% (2)	14.3% (3)	19.0% (4)	12.3 ± 7.2
Total sample	38.6% (163)	41.2% (174)	44.8% (189)	25.46 ± 14.82
Statistical test	*χ*^2^ = 48.36, *p* < 0.001	*χ*^2^ = 42.18, *p* < 0.001	*χ*^2^ = 51.74, *p* < 0.001	*F* = 36.92, *p* < 0.001

**Figure 5:**
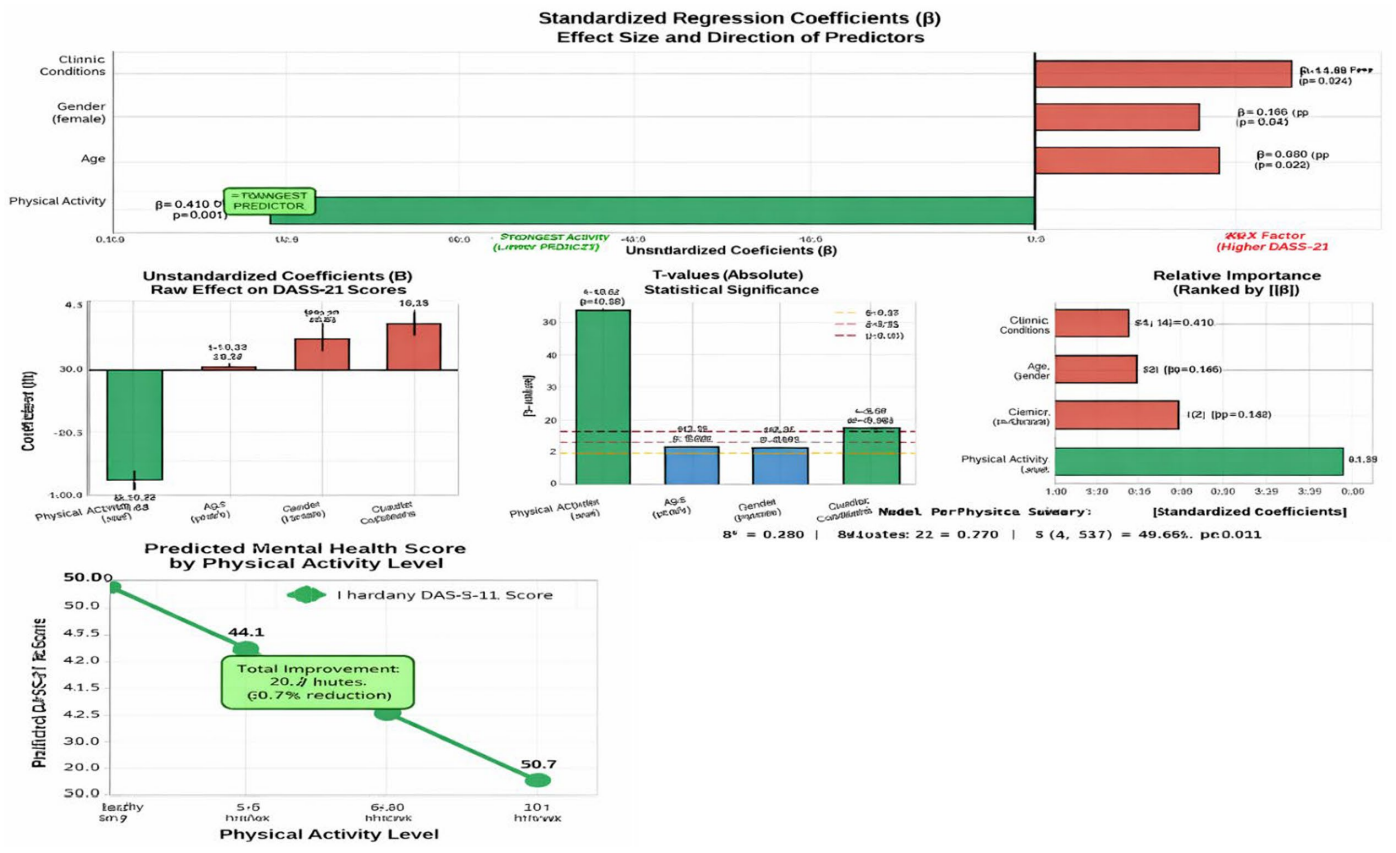
Standardized (*β*) and unstandardized (*B*) regression coefficients of predictors of DASS-21 total score, indicating the direction and the strength of the effect of each predictor on mental health outcomes.

The regression analysis in Table 5, Figure 5 indicated that the level of physical activity is an important negative predictor of psychological distress: on average, the total scores of the DASS-21 were reduced by 6.72 points with each gain in the level of the activity category (*β* = −0.41, *p* < 0.001), even within the adjusted sample in terms of age, gender, or chronic diseases. This means that older adults who were more physically active had been found to have much better mental health. Moreover, mental health was worse in the case of having chronic conditions (*β* = 3.15, *p* < 0.001), and older age and gender (female) had less significant but still significant effects. The model was generally statistically significant (*F* = 31.28, *p* < 0.001), and it explained 23% of the variation in mental health outcomes, which supports the key position of physical activity as a partially modifiable protective factor of mental health in late life.

**Table 5:** Multiple linear regression analysis predicting mental health (DASS-21 total score) from physical activity and covariates (*N* = 422).

Predictor	Unstandardized coefficient (*B*)	Std. error	Standardized coefficient (*β*)	*t*	*p*-Value
(Constant)	36.84	2.15	—	17.14	<0.001
Physical activity level 1 = hardly any, 2 = 1–5 h, 3 = 5–10 h, 4 = 10+ h	−6.72	0.63	−0.41	10.67	<0.001
Age (years)	0.28	0.12	0.09	2.33	0.020
Gender (0 = male, 1 = female)	1.94	0.86	0.08	2.26	0.024
Chronic conditions (0 = none, 1 = ≥1)	3.15	0.92	0.14	3.42	<0.001

The prediction equation is


$$\begin{array}{l} \text{DASS}-21\;\text{Total Score}=36.84+(-6.72\times \text{Physical Activity Level})+\\ \;(0.28\times \mathrm{Age})+(1.94\times \text{Gender})+\\ \;(3.15\times \text{Chronic Conditions}) \end{array}$$

## 4 Discussion

The results of the study give an in-depth multi-faceted image of the connection between physical activity and psychological health among older adults people that belong to sports clubs. Not only is this statistically significant, it means that there is in fact some sort of protection and curative effect, as a form of movement is the non-pharmaceutical healer, which will make the mind get on track again and make a person feel a part of something competent. People who exercise 10 h or above every week, are not only living a healthier life but also have a calm mind, evidenced by their DASS-21 scores of 12.3 which is within the normal range of scores and does not depict any clinical disease. On the other hand, one out of every three samples of the population is practically completely physically inactive, and in that instance, they have high levels of psychological distress (mean DASS = 34.2) which is higher than the moderate level of severity of any of the dimensions of the scale. The increased body of evidence regarding the significance of physical activity in older people indicates the importance of this topic. The recent research by Karuna Moorthi et al. (28), Hou et al. (29), and Meng et al. (13) continues to depict the close relationship between frequent physical activity and improved psychological health, especially among older adults who attend sports clubs. On top of its physiological impacts, it seems that physical activity enhances mental well-being, promoting social competence and interpersonal relationships, which leads to an improved overall quality of life, which is one of the essential elements of healthy aging (13, 29). This is consistent with the results of Althumiri et al. (30), who have found that moderate-intensity physical activity follows the recommendations of the World Health Organization, and it is significantly related to a lower risk of major depressive disorder and generalized anxiety disorder among adults.

The physical activity behaviors of older adults show that they have a high preference for or preference towards low-risk, social activities familiar to the cultural movement, and the activity most used is walking; more than 92% of the sample reported being engaged in walking. This is in line with bigger gerontological literature, which emphasizes walking as not only a safe and convenient exercise but also as a social channel connecting and establishing a routine. In this respect, one can see in the findings that the social relationships were found to be the best domain of quality of life among older adults (M = 3.70), although 60 percent of their sample were physically inactive, and the overall quality of life was low (36%), as study by Ngah and Lian (10) in Malaysia. This implies that although social embeddedness is very well appreciated in aging populations in both Southeast Asia and the Gulf, it does not always translate into physical interaction. With relatively high sports club enrolment numbers, 30.8% of older adults reported that they participated in almost no exercise, which is a paradoxical situation that demonstrates a significant disconnect between the capacity and utilization of facilities (28). This means that access to infrastructure is not enough, but customized and motivation-receptive programming is needed. The above interventions have shown that group-based programs (especially those that are supervised) of structured walking are effective in improving physical functioning and psychosocial well-being in sedentary older adults (24). These interventions will not only deal with physical inactivity but also reverse social isolation by integrating movement into the daily activities of the community.

In addition, the uptake of active lifestyles by older people is complicated by a combination of several factors, such as the presence of chronic illnesses, the accessibility of recreational facilities that are age-specific, and the intensity of social support (31). The combination of these determinants determines the viability and the sustainability of physical activities in the later years of life. Unless these multilevel barriers are tackled, there is a likelihood that the process of public health will not achieve all its objectives. Thus, as Al-Nozha et al. (32) stressed more than 10 years ago, and the existing evidence confirms, the ability of physical activity to become a building block of healthy aging is achievable with comprehensive, context-sensitive practices that will enable physical activity to become not another underutilized opportunity but a pillar of well-being in the aging population of the country.

### 4.1 Study strengths and limitations

There are several strengths that can be noticed in this study that can contribute to the validity and relevance of the findings. To start with, it offers empirical evidence of the Egyptian context, which is presented in rare research on mental health and physical activity interaction in older adults, a group that is not yet represented in research on the health of the region. Second, the application of the validated Arabic version of the DASS-21 and Rapid Assessment of Physical Activity (RAPA) guaranteed the reliability and cultural suitability in the measurement of the two types of constructs, as well as the psychological and behavioral ones. Third, the size of the sample (422) used in the study is rather large; it was taken across various sports clubs in El Minya, which increases the statistical power and applicability of findings in the study to other similar community-based settings in Egypt. Lastly, the use of strong analytical tools, such as the multiple linear regression model that accounts for the presence of important covariates (age, gender, chronic conditions), enhances causal inference of the protective effect of physical activity on psychological distress.

However, several limitations should be taken into consideration. To begin with, the research design was cross-sectional and cannot make conclusive findings regarding causality. Whereas the data provided indicate that physical activity correlates with an improved mental health state, longitudinal research is necessary to define the directionality of the given correlation. Second, the sample of the study was restricted to older adults already playing in sports clubs, which has created the risk of selection bias: such participants are probably healthier, more physically fit, and more socially active than those who do not attend sports clubs. This prior activity in sports clubs might have bestowed some pre-existing physical adaptations of higher aerobic capacity and muscular strength that might have mitigated the observed positive effects of physical activity on mental health, thus over-estimating the level of activity and under-estimating the level of psychological distress compared to the general older adults population. To determine the actual effects of structured intervention in physical activity, future studies should make use of a comparative design where older people who attend fitness centers are compared to a similar group of physically inactive individuals. The benefit of such an approach would be to identify whether the benefits observed are as a result of physical activity, or a synergistic effect of access to facilities, social support, and habitual participation. Third, physical activity was measured through self-report, and it could be affected by recall or social desirability bias, even though a validated measure (RAPA) was used. Further research would give more accurate results on objective measures (e.g., accelerometers). Fourth, although chronic conditions were considered, the severity of diseases, taking of medications, and cognitive functions were not evaluated in the study, and these factors may affect both activity level and mental well-being. Finally, the research results might not be generalized to older adults people in rural areas or those with severe functional impairment who cannot participate in club-based intervention programs.

## 5 Conclusion

This research gives strong reasons to believe that exercise is a powerful, non-pharmacological treatment method for mental health and quality of life improvement among the older adults population. The findings demonstrate a good, dose-dependent inverse relationship between the amount of psychological distress and the amount of movement: older adults who had more movement particularly 10 or more hours per week had DASS-21 scores squarely within the normal range, and those who were largely inactive were also discovered to possess huge volumes of depression, anxiety, and stress. This gradients effect implies that movement is not only beneficial, but also a protective variable; this fact is the one that indicates that the movement is a global phenomenon and the supporting factor of movement as a therapeutic paradigm in a culturally specific Arab context. However, the activities that the participant prefers to participate in, i.e., walking, light aerobics, and flexibility exercises are the ones that have been chosen wisely based on the safety, accessibility, and integration into the social community and is reflected in both the quality of life and the older generation in general.

In addition, the findings of regression analysis confirmed the fact that the level of physical activity is the most important factor that can be altered and is a better predictor of mental well-being compared to the other factors including age, gender, or even the weight of chronic diseases. This is a position that uses physical activity as a foundation of healthy aging in a squarely consistent manner with the Saudi Vision 2030 objectives of improving dignified, active, and socially inclusive later life. In accordance with these outcomes, policymakers, medical practitioners, and populations must collaborate in order to integrate culturally tailored physical exercise programs into primary care and geriatric facilities. Conclusively, this paper confirms that movement is not just exercise, but it is medicine to the mind and the soul. Through the adoption of a strategy of movement as a form of therapy, Egypt can make aging a resilient, bonding, and healthy experience.

## Data Availability

The original contributions presented in the study are included in the article/Supplementary material, further inquiries can be directed to the corresponding author.
