# Supplementary material for: Movement as therapy an analytical study of the role of physical activity in building strong mental health for older adults
Source: Front Public Health. 2026 Apr 10;14:1797406. doi: 10.3389/fpubh.2026.1797406 (PMC13107243; doi:10.3389/fpubh.2026.1797406)
Supplement: Supplementary file 1 [file Supplementary_file_1.docx]

**Appendix A: Physical Activity Assessment Instrument (Adapted RAPA of Egyptian Older Adults)**

**Part 1: Aerobic and General Physical Activity**

Please indicate how often you engaged in the following over the past month:

Light physical activity (e.g., slow walking, light housework) for at least 10 minutes at a time:

☐ Never / Rarely

☐ 1–2 days/week

☐ 3–4 days/week

☐ 5+ days/week

- If 3–5 days/week or more, how many total hours per week? ______ hours

Moderate physical activity (e.g., brisk walking, light aerobics, gardening) that increases your breathing or heart rate:

☐ Never / Rarely

☐ 1–2 days/week

☐ 3–4 days/week

☐ 5+ days/week

- If 3–5 days/week or more, how many total hours per week? ______ hours

Vigorous physical activity (e.g., running, fast cycling, swimming laps):

☐ Never / Rarely

☐ 1–2 days/week

☐ 3+ days/week

- If yes, how many total hours per week? ______ hours

**Part 2: Strength and Flexibility**

Do you do activities to strengthen your muscles (e.g., lifting weights, resistance bands, bodyweight exercises) at least 2 days per week?

☐ Yes ☐ No

Do you do activities to improve balance or flexibility (e.g., stretching, yoga, Tai Chi) at least 2 days per week?

☐ Yes ☐ No

**Part 3: Activity Type Checklist**

(Please check all that apply)

☐ Walking

☐ Light aerobic exercises (e.g., chair-based workouts, low-impact dance)

☐ Stretching / Flexibility exercises

☐ Running or jogging

☐ Cycling (indoor or outdoor)

☐ Swimming

☐ Strength training

☐ Culturally based movement (e.g., light farming, prayer-related motion)

☐ Other: _______________

**Scoring Procedure Used in This Study**

Total weekly physical activity duration was calculated by summing estimated hours from all reported activities.

Participants were then classified into four categories for analysis:

Hardly any: < 1 hour/week

1–5 hours/week

5–10 hours/week

10+ hours/week

**Appendix B:**

**S1:DASS-21 scales among Older Adults**

| **No.** | **Statement** | ***Did not apply at all*** | ***Applied slightly*** | ***Applied moderately*** | ***Applied considerably*** | ***Applied very much*** |
| --- | --- | --- | --- | --- | --- | --- |
| **Depression** | | | | | | |
| 1 | I couldn’t seem to experience any positive feeling |  |  |  |  |  |
| 2 | I found it difficult to work up the initiative to do things |  |  |  |  |  |
| 3 | I felt that I had nothing to look forward to |  |  |  |  |  |
| 4 | I felt down-hearted and blue |  |  |  |  |  |
| 5 | I was unable to become enthusiastic about anything |  |  |  |  |  |
| 6 | I felt I wasn’t worth much as a person |  |  |  |  |  |
| 7 | I felt that life was meaningless |  |  |  |  |  |
| **Anxiety** | | | | | | |
| 8 | I was aware of dryness of my mouth |  |  |  |  |  |
| 9 | I experienced breathing difficulty |  |  |  |  |  |
| 10 | I experienced trembling (e.g., in the hands) |  |  |  |  |  |
| 11 | I was worried about situations in which I might make a fool of myself |  |  |  |  |  |
| 12 | I felt I was close to panic |  |  |  |  |  |
| 13 | I was aware of the action of my heart |  |  |  |  |  |
| 14 | I felt scared without any good reason |  |  |  |  |  |
| **Stress** | | | | | | |
| 15 | I tended to over-react to situations |  |  |  |  |  |
| 16 | I felt that I was using a lot of nervous energy |  |  |  |  |  |
| 17 | I found myself getting agitated |  |  |  |  |  |
| 18 | I found it difficult to relax |  |  |  |  |  |
| 19 | I was intolerant of anything |  |  |  |  |  |
| 20 | I felt that I was rather touchy |  |  |  |  |  |
| 21 | I tended to over-react to situations |  |  |  |  |  |

**Table S1. Frequency Distribution of Responses to Stress Subscale Items of DASS-21 among Older Adults (N = 422)**

| **Statement** | ***Did not apply at all*** | ***Applied slightly*** | ***Applied moderately*** | ***Applied considerably*** | ***Applied very much*** | **Mean** | **SD** | **X^2^** | **Sig.** | **Impact %** |
| --- | --- | --- | --- | --- | --- | --- | --- | --- | --- | --- |
| **Depression** |  |  |  |  |  |  |  |  |  | KMO.0.842 |
| 1 | 198 (46.9%) | 124 (29.4%) | 68 (16.1%) | 22 (5.2%) | 10 (2.4%) | 0.92 | 0.88 | 112.34 | <0.001 | 23.7% |
| 2 | 142 (33.6%) | 156 (37.0%) | 82 (19.4%) | 30 (7.1%) | 12 (2.8%) | 1.18 | 1.02 | 94.76 | <0.001 | 29.4% |
| 3 | 210 (49.8%) | 132 (31.3%) | 54 (12.8%) | 18 (4.3%) | 8 (1.9%) | 0.84 | 0.91 | 132.51 | <0.001 | 19.0% |
| 4 | 196 (46.4%) | 128 (30.3%) | 62 (14.7%) | 26 (6.2%) | 10 (2.4%) | 0.96 | 0.94 | 108.29 | <0.001 | 23.2% |
| 5 | 204 (48.3%) | 130 (30.8%) | 56 (13.3%) | 20 (4.7%) | 12 (2.8%) | 0.90 | 0.93 | 119.87 | <0.001 | 20.8% |
| 6 | 238 (56.4%) | 108 (25.6%) | 50 (11.8%) | 16 (3.8%) | 10 (2.4%) | 0.72 | 0.85 | 156.42 | <0.001 | 17.6% |
| 7 | 242 (57.3%) | 102 (24.2%) | 48 (11.4%) | 18 (4.3%) | 12 (2.8%) | 0.70 | 0.87 | 160.05 | <0.001 | 18.5% |
| Total |  |  |  |  |  | 8.42 | 5.73 | 184.32 | <0.001 | 38.6% |
| **Anxiety** |  |  |  |  |  |  |  |  |  | KMO. 0.827 |
| 8 | 174 (41.2%) | 132 (31.3%) | 76 (18.0%) | 26 (6.2%) | 14 (3.3%) | 1.04 | 1.01 | 88.63 | <0.001 | 27.5% |
| 9 | 182 (43.1%) | 120 (28.4%) | 74 (17.5%) | 30 (7.1%) | 16 (3.8%) | 1.02 | 1.05 | 97.28 | <0.001 | 28.4% |
| 10 | 224 (53.1%) | 112 (26.5%) | 52 (12.3%) | 22 (5.2%) | 12 (2.8%) | 0.78 | 0.95 | 142.19 | <0.001 | 20.4% |
| 11 | 192 (45.5%) | 130 (30.8%) | 62 (14.7%) | 24 (5.7%) | 14 (3.3%) | 0.94 | 0.98 | 103.74 | <0.001 | 23.7% |
| 12 | 228 (54.0%) | 110 (26.1%) | 50 (11.8%) | 20 (4.7%) | 14 (3.3%) | 0.76 | 0.93 | 148.56 | <0.001 | 19.7% |
| 13 | 202 (47.9%) | 122 (28.9%) | 60 (14.2%) | 24 (5.7%) | 14 (3.3%) | 0.88 | 0.96 | 124.33 | <0.001 | 23.2% |
| 14 | 232 (55.0%) | 106 (25.1%) | 52 (12.3%) | 18 (4.3%) | 14 (3.3%) | 0.74 | 0.92 | 151.08 | <0.001 | 19.9% |
| Total |  |  |  |  |  | 7.89 | 5.21 | 203.17 | <0.001 | 41.2% |
| **Stress** |  |  |  |  |  |  |  |  |  | KMO. 0.851 |
| 15 | 156 (37.0%) | 140 (33.2%) | 74 (17.5%) | 34 (8.1%) | 18 (4.3%) | 1.28 | 1.09 | 76.41 | <0.001 | 39.8% |
| 16 | 162 (38.4%) | 136 (32.2%) | 70 (16.6%) | 32 (7.6%) | 22 (5.2%) | 1.24 | 1.07 | 82.15 | <0.001 | 38.4% |
| 17 | 190 (45.0%) | 128 (30.3%) | 64 (15.2%) | 24 (5.7%) | 16 (3.8%) | 1.00 | 0.99 | 110.67 | <0.001 | 24.7% |
| 18 | 168 (39.8%) | 132 (31.3%) | 72 (17.1%) | 30 (7.1%) | 20 (4.7%) | 1.16 | 1.04 | 89.38 | <0.001 | 33.2% |
| 19 | 186 (44.1%) | 130 (30.8%) | 62 (14.7%) | 26 (6.2%) | 18 (4.3%) | 1.06 | 1.00 | 101.92 | <0.001 | 25.2% |
| 20 | 208 (49.3%) | 118 (28.0%) | 56 (13.3%) | 22 (5.2%) | 18 (4.3%) | 0.92 | 0.97 | 127.84 | <0.001 | 22.7% |
| 21 | 156 (37.0%) | 140 (33.2%) | 74 (17.5%) | 34 (8.1%) | 18 (4.3%) | 1.28 | 1.09 | 76.41 | <0.001 | 39.8% |
| Total |  |  |  |  |  | 9.15 | 6.04 | 218.65 | <0.001 | 44.8% |
| Total DASS |  |  |  |  |  | 25.46 | 14.82 | 606.14 | <0.001 | KMO. 0.851 |
